# Tocotrienol-Rich Tocomin Attenuates Oxidative Stress and Improves Endothelium-Dependent Relaxation in Aortae from Rats Fed a High-Fat Western Diet

**DOI:** 10.3389/fcvm.2016.00039

**Published:** 2016-10-17

**Authors:** Saher F. Ali, Jason C. D. Nguyen, Trisha A. Jenkins, Owen L. Woodman

**Affiliations:** ^1^School of Health and Biomedical Sciences, RMIT University, Bundoora, VIC, Australia

**Keywords:** tocotrienol, endothelium, oxidative stress, high-fat diet, vascular relaxation

## Abstract

We have previously reported that tocomin, a mixture high in tocotrienol content and also containing tocopherol, acutely preserves endothelial function in the presence of oxidative stress. In this study, we investigated whether tocomin treatment would preserve endothelial function in aortae isolated from rats fed a high-fat diet known to cause oxidative stress. Wistar hooded rats were fed a western diet (WD, 21% fat) or control rat chow (standard diet, 6% fat) for 12 weeks. Tocomin (40 mg/kg/day sc) or its vehicle (peanut oil) was administered for the last 4 weeks of the feeding regime. Aortae from WD rats showed an impairment of endothelium-dependent relaxation that was associated with an increased expression of the NADPH oxidase Nox2 subunit and an increase in the vascular generation of superoxide measured using L-012 chemiluminescence. The increase in vascular oxidative stress was accompanied by a decrease in basal NO release and impairment of the contribution of NO to ACh-induced relaxation. The impaired relaxation is likely contributed to by a decreased expression of eNOS, calmodulin, and phosphorylated Akt and an increase in caveolin. Tocotrienol rich tocomin, which prevented the diet-induced changes in vascular function, reduced vascular superoxide production and abolished the diet-induced changes in eNOS and other protein expression. Using selective inhibitors of nitric oxide synthase (NOS), soluble guanylate cyclase (sGC) and calcium-activated potassium (K_Ca_) channels we demonstrated that tocomin increased NO-mediated relaxation, without affecting the contribution of endothelium-dependent hyperpolarization type relaxation to the endothelium-dependent relaxation. The beneficial actions of tocomin in this diet-induced model of obesity suggest that it may have potential to be used as a therapeutic agent to prevent vascular disease in obesity.

## Introduction

The increasing incidence of obesity in western societies is clearly correlated with the incidence of cardiovascular disease (1). There is growing evidence that a contributing mechanism to the development of obesity-induced cardiovascular disease is an associated increase in oxidative stress (2). A well-characterized trigger for the initiation and progression of vascular disease is an impairment of endothelial function. Feeding rats and mice a “western” diet (WD), that is, a diet with an elevated level of total and saturated fats, leads to impairment of endothelium-dependent relaxation (3–5). The impairment of endothelium-dependent relaxation is strongly linked to obesity-induced increases in oxidative stress due to increased synthesis of superoxide anions in the vasculature.

There has been considerable interest in the potential use of vitamin E to reduce cardiovascular disease but largely with disappointing outcomes from clinical trials [for review, see Ref. (6, 7)]. Most clinical trials have focused specifically on the actions of α-tocopherol, the vitamin E form found most abundantly in mammalian tissues (8), but vitamin E has three additional isoforms of tocopherol (β, γ, δ) and four tocotrienols (α, β, γ, δ). There is growing evidence that tocotrienols may exert biological activities similar to, but also distinct from, the tocopherols (9, 10). When investigating the capacity of tocotrienols to preserve endothelial function in the presence of oxidative stress *in vitro* we found that when present as individual isomers α-, δ-, and γ-tocotrienol failed to demonstrate any beneficial effect (11). Importantly, this lack of preservation of endothelium-dependent relaxation was despite the demonstration of effective antioxidant activity in a separate assay. This contrasted with the ability of tocomin, a palm oil extract containing a high concentration of tocotrienols and a lesser component of tocopherol, to significantly improve endothelium-dependent relaxation in the presence of oxidative stress. Crucially, the protective actions of tocomin could be replicated by a mixture of tocotrienols with, but not without, tocopherol. Thus, we came to the conclusion that a mixture of tocotrienols and tocopherol may provide more effective protection of endothelial function against oxidative stress than any of the individual components in isolation. Therefore the aim of the present study is to investigate whether the acute protective actions of tocomin can be replicated when subcutaneously administered *in vivo* to rats where oxidative stress is induced by consumption of a high-fat WD that causes endothelial dysfunction.

## Materials and Methods

### Animals

All procedures were approved by the Animal Experimentation Ethics Committee of RMIT University and conformed to the National Health and Medical Research Council of Australia code of practice for the care and use of animals for scientific purposes.

### Western Diet Feeding Regime

Male Wistar Hooded rats (University of Adelaide, Australia) weighing 180–200 g at the start of the feeding period were housed in groups of four under a light/dark cycle (12/12 h), in a temperature controlled room (22°C) at the RMIT Animal Facility with water *ad libitum*. Animals were randomly assigned to either a standard (control) diet (SD, Standard AIN93G rodent diet, 7% total fat including 1.05% total saturated fatty acids; Specialty Feeds, Perth, Australia) or WD (SF00-219, 21% total fat including 1.80% total saturated fats and 0.15% cholesterol; Specialty Feeds, Perth, Australia). Animals were allowed *ad libitum* access to their designated diets for 12 weeks. Food intake and bodyweight were measured weekly. At the end of this period, rats were killed using asphyxiation by CO_2_ inhalation, followed by decapitation, and their chests were opened to isolate the thoracic aortae. Non-fasted blood samples were obtained from the carotid arteries following decapitation. Blood glucose levels were measured using a one-touch glucometer (Roche, Sydney, NSW, Australia). HbA1c was also measured using the In2it™ A1c system (II) analyzer (Bio-Rad, Hercules, CA, USA).

### Drug Administration

Eight weeks after commencement of the feeding period, tocomin (40 mg/kg/day sc) or vehicle (peanut oil) was administered for a period of 4 weeks until cessation of the study. We chose to deliver tocomin subcutaneously to ensure accuracy of dose delivery to each rat rather than by inclusion in the diet and therefore the oral route. Tocomin (Carotech, Malaysia) is a palm oil extract containing a tocotrienol-rich fraction (40%), palm olein (38%), and α-tocopherol (11%).

### Assessment of Vascular Function

The thoracic aorta was isolated and immediately placed in ice-cold Krebs-bicarbonate solution (118 mM NaCl, 4.7 mM KCl, 1.18 mM MgSO_4_, 1.2 mM KH_2_PO_4_, 25 mM NaHCO_3_, 11.1 mM d-glucose, and 1.6 mM CaCl_2_). The aorta was then cleared of fat and connective tissue and cut into 2- to 3-mm long segments. The aortic rings were mounted between two stainless steel wires, one of which was linked to an isometric force transducer (model FT03, Grass Medical Instruments, Quincy, MA, USA) connected to a Powerlab (model 8/30 AD Instrument Co., Sydney, Australia), and the other end anchored to a glass rod submerged in a standard 10-mL organ bath. The organ bath was filled with Krebs-bicarbonate solution. The bath medium was maintained at 37°C, pH 7.4, and continuously aerated with 95% O_2_ and 5% CO_2_. Aortic rings were equilibrated for 45 min at a resting tension of 1 g and then were contracted with an isotonic, high potassium physiological salt solution (KPSS, 122.7 mM KCl, in which K^+^ ions replaced Na^+^ ions in the solution) for 20 min to achieve maximal contraction. The KPSS was then replaced with Krebs solution to allow aortic segments to re-equilibrate, and the rings were then sub-maximally contracted with phenylephrine (PE) to 40–60% of KPSS contraction (PE, 0.01–0.3 μM). Endothelial integrity was tested by the addition of a single concentration of acetylcholine (ACh, 10^−5^M). Where relaxation was greater than 80% of the pre-contraction, the endothelium was considered to be intact and the aortic ring was included in the study. Some additional segments of the thoracic aortae were used to measure superoxide production (see below).

Cumulative concentration–response curves to ACh (0.1 nM–0.1 mM) and sodium nitroprusside (SNP, 0.1 nM–0.1 mM) were determined using aortic rings contracted with PE (10^−8^–10^−7^M) to 40–60% of maximal contraction. Responses to ACh and SNP were also tested in the presence or absence of a small potassium activated calcium channel inhibitor (SK_Ca_, apamin, 1 μM), intermediate potassium-activated calcium channel inhibitor (IK_Ca_, TRAM-34, 1 μM), nitric oxide synthase (NOS) inhibitor, N-nitro-l-arginine methyl ester (L-NAME, 100 μM), and soluble guanylate cyclase (sGC) inhibitor, 1H-[1,2,4]oxadiazolo[4,3-a]quinoxalin-1-one (ODQ, 10 μM) to investigate the role of NO and endothelium-dependent hyperpolarization (EDH) through the opening of potassium channels in the relaxant responses. All treatments were added to the baths 20 min prior to conducting the concentration–response curves. The negative logarithm of the concentration at which 50% relaxation occurred (pEC_50_), and maximum relaxation (*R*_max_) values were calculated from the individual cumulative concentration response curves using Graphpad Prism 6.

### Basal Nitric Oxide Activity

Following maximal contraction of the aortae with KPSS, the tissues were washed and relaxed to basal tension and then pre-contracted with PE (10–100 nM) to approximately 20–30% of the maximal KPSS contraction. Under those conditions the further addition of l-NAME (100 μM), a NOS inhibitor, causes further contraction the level of which correlates with the level of the basal release of NO (12, 13).

### Superoxide Production in the Aorta

Superoxide production in aortic rings was measured using L-012 chemiluminescence. The assay is based on methods developed by Miller et al. (14) with the following modifications. Aortic segments were cleared of fat and connective tissue and cut into 2–3 mm long segments, which were incubated at 37°C for 30 min in Krebs-HEPES buffer either alone or in the presence of apocynin (300 μM), a non-specific NADPH oxidase (Nox) inhibitor, in a cell culture plate. Krebs-HEPES buffer (300 mL), containing L-012 (100 mM, Wako Pure Chemicals, Osaka, Japan) and the appropriate treatments were placed in a 96-well optiplate, which was loaded into a Polarstar Optima plate reader (BMG Labtech, Melbourne, VIC, Australia) to measure background photon emission at 37°C. After background reading was completed, a single aortic segment was added to each well in the optiplate and photon emission was recounted. Superoxide production was quantified by subtracting the final reading from the background reading, and counts were then taken as arbitrary units of superoxide production and expressed as a ratio to dry tissue mass (AU/mg dry tissue).

### Protein Expression

Western blots were performed as described previously (14, 15) with the following modifications. The tissues were homogenized in 200 mL of ice-cold lysis buffer [100 mM NaCl, 10 mM Tris, 2 mM EDTA, 0.5% w/v sodium deoxycholate, 1% vol/vol Triton X-100, pH 7.4, protease, and phosphatase inhibitor cocktails (Roche, Sydney, NSW, Australia)], and the total protein concentration of the samples was quantified using the Bio-Rad Bradford assay. Protein samples were thawed and heated at 95°C for 5 min. Equal amounts of protein homogenate (30 μg) were loaded onto the SDS-PAGE gels and ran at 100 V until the lowest molecular weight marker was at the bottom of the gel. Proteins in the SDS gel were transferred onto a nitrocellulose membrane (0.45 μm pore size) using a wet transfer at 90 V for 90 min at 4°C. Membranes were blocked in 0.25% BSA in tris-base saline Tween 20 (TBST pH 7.4) for 1 h at room temperature and incubated with anti-mouse/rabbit primary antibodies probing for proteins of interest (eNOS, Nox2, and caveolin-1, BD Transduction Laboratories, Lexington, KY, USA; Akt and pAkt, Cell Signaling, Danvers, MA, USA; calmodulin, Merck, Millipore, Australia; all antibody dilutions were 1:1000 in TBST, overnight, 4°C). Membranes were washed the following the day (3 × 10 min) in TBST followed by incubation in HRP-linked mouse/rabbit secondary antibody for 1 h at room temperature. Membranes were washed and then developed using a ChemiDoc XRS (Bio-Rad, Sydney, NSW, Australia). All proteins were detected using either enhanced chemiluminescence (Amersham, GE Healthcare, Sydney, NSW, Australia) or Supersignal West Femto (Thermo Scientific, Rockford, IL, USA). Membranes were then blocked in 0.1% sodium azide (HRP inhibitor) for 1 h at room temperature and then re-probed with the loading control primary antibody [anti-mouse/rabbit β-actin (1:2000)] as described above.

### Statistics

All results are expressed as mean ± SEM, where *n* represents the number of animals per group. Concentration–response curves from the rat-isolated aortae were constructed and fitted to a sigmoidal curve using non-linear regression (Graphpad Prism version 6.0, Graphpad Software, San Diego, CA, USA) to calculate the sensitivity of each agonist (pEC_50_). Maximum relaxation (*R*max) to ACh was measured as a percentage of the precontraction to PE. Group pEC_50_ and *R*max values were compared using a two-way ANOVA with *post hoc* analysis using Dunnett’s test as appropriate. *p* < 0.05 was considered statistically significant.

l-NAME-induced contraction, representing basal NO release, was expressed as a percentage of the KPSS-induced contraction ± SEM. The values were compared using a two-way ANOVA with *post hoc* analysis using Dunnett’s test. *p* < 0.05 was considered statistically significant. Superoxide levels from rat aortic rings are expressed as average counts per second ± SEM normalized to dry tissue weight. Results were compared by two-way ANOVA with a *post hoc* Dunnett’s test. *p* < 0.05 was considered statistically significant.

All western blotting results were quantified by densitometry using the ImageLab software (Bio-Rad, Sydney, NSW, Australia) and expressed as a densometric ratio of the primary protein to β-actin ± SEM. The quantification for the expression of pAkt is expressed as a ratio of pAkt to Akt ± SEM. Results were compared by two-way ANOVA with a *post hoc* Dunnett’s test. *p* < 0.05 was considered statistically significant.

## Results

### Body Weights and Blood Glucose

The body weight of the WD fed rats was significantly greater than that of the SD fed rats at the end of the experimental period. The final bodyweight of the tocomin treated WD rats was not significantly different to the WD fed rats without tocomin. The epididymal fat mass was significantly increased in the WD fed rats, with or without tocomin treatment, compared to the SD fed rats (Table 1).

**Table 1:** **Mean body weight, blood glucose, HbA1c, and epididymal fat mass at the end of the experiment of standard diet (SD) and western diet (WD) rats**.

	*n*	SD	*n*	WD	*n*	SD + tocomin	*n*	WD + tocomin
Final body weight (g)	10	415 ± 10	10	456 ± 10*	10	421 ± 9	10	440 ± 8
Blood glucose (mmol/L)	9	8.4 ± 1	10	8.6 ± 0.9	9	8.8 ± 1	10	9.0 ± 1.3
HbA1c (%)	9	5.4 ± 0.3	10	5.2 ± 0.1	8	6.2 ± 0.3	10	5.9 ± 0.4
Epididymal fat (g)	10	8.5 ± 0.8	10	12.9 ± 1.2*	10	9.8 ± 1	10	12.4 ± 0.9*

*Significantly different to SD, p < 0.05.

*Two-way ANOVA Dunnett’s multiple comparison test*.

The blood glucose and glycated hemoglobin (HbA1c) levels were not significantly affected by the WD or by 4-week tocomin treatment in either WD or SD fed rats (Table 1).

### Superoxide Production

**Figure 1:**
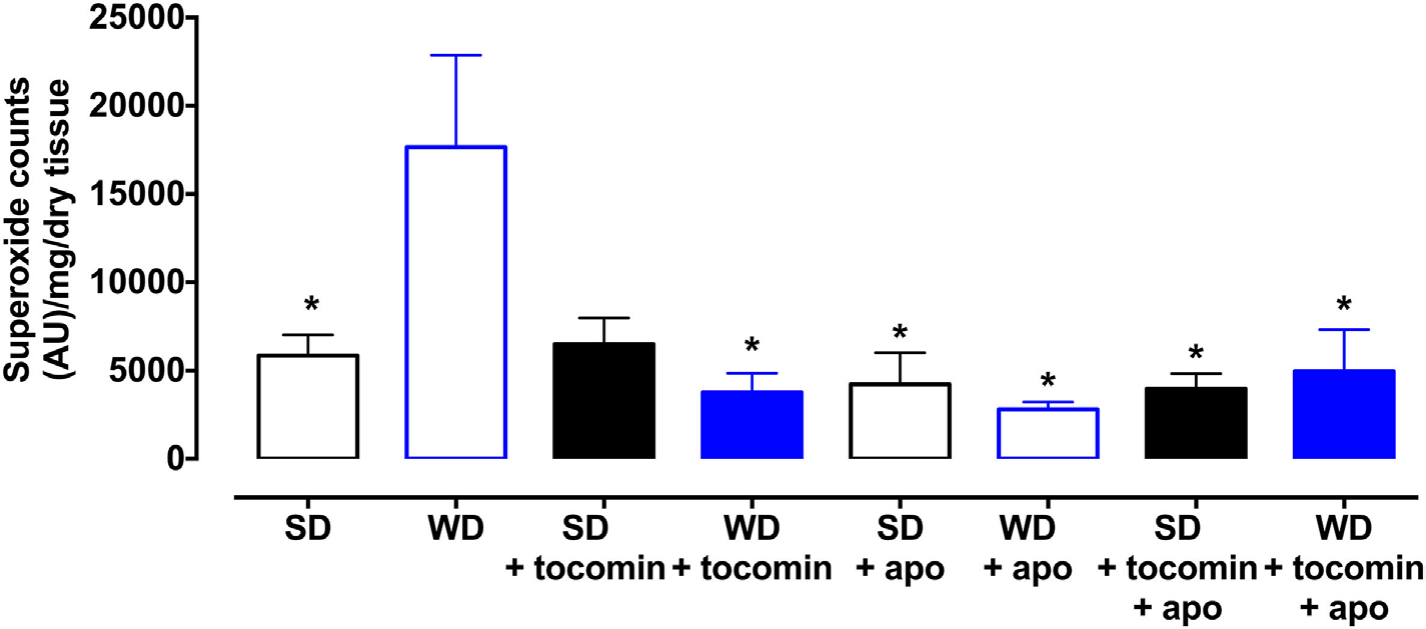
**Superoxide generated in rat aorta in the presence of NADPH from standard diet (SD), western diet (WD), and tocomin treated (SD + tocomin/WD + tocomin) groups in the absence and presence of apocynin (300 μM)**. Data are expressed as mean ± SEM. *Significantly different to WD *p* ≤ 0.05, two-way ANOVA, Dunnett’s multiple comparisons test. *n* = 3–6.

Basal superoxide production was significantly elevated in aortae from rats fed the WD in comparison to the SD rat aortae. The 4-week treatment with tocomin (40 mg/kg/day sc) significantly attenuated superoxide production by the aortae from a WD fed rat without affecting superoxide production by the SD rat aortae (Figure 1). Treatment with apocynin, a non-selective inhibitor of NADPH oxidase, decreased superoxide levels in the aorta from WD fed rats.

### Basal Nitric Oxide Levels and the Effect of Tocomin

**Figure 2:**
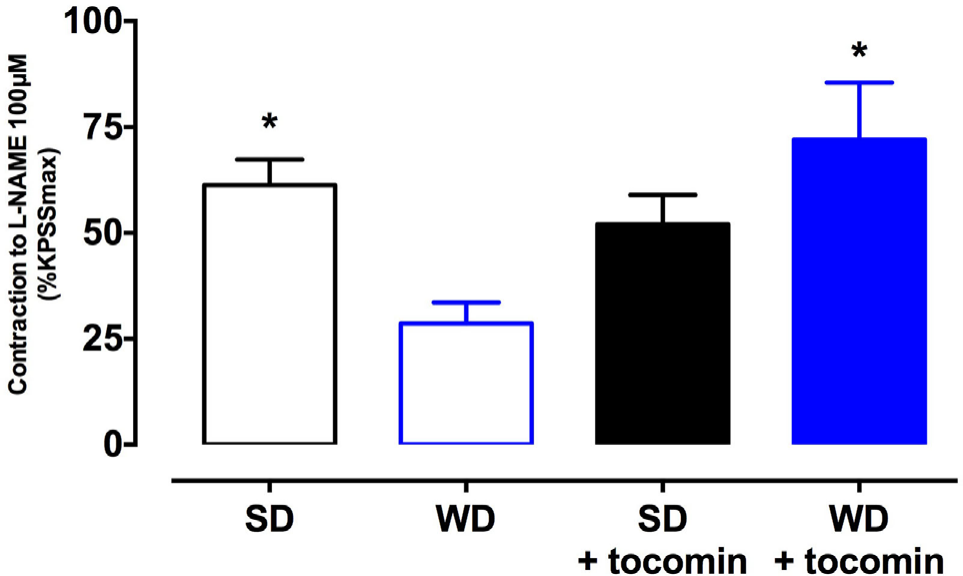
**Response to l-NAME in the presence of KPSS (SD), western diet (WD), and tocomin-treated (SD + tocomin/WD + tocomin) groups**. Data are expressed as mean ± SEM. *Significantly different to WD. *p* ≤ 0.05. *n* = 10, Dunnett’s multiple comparisons test. *n* = 3–6.

The contractile response of the aortae to the presence of the eNOS inhibitor l-NAME was significantly less in the aortae from WD rats compared to the SD rat aorta (Figure 2). The 4-week treatment of the WD rats with tocomin significantly improved the contractile response to l-NAME indicating increased basal NO synthesis.

### Effect of Western Diet and Tocomin on Vascular Relaxation

**Figure 3:**
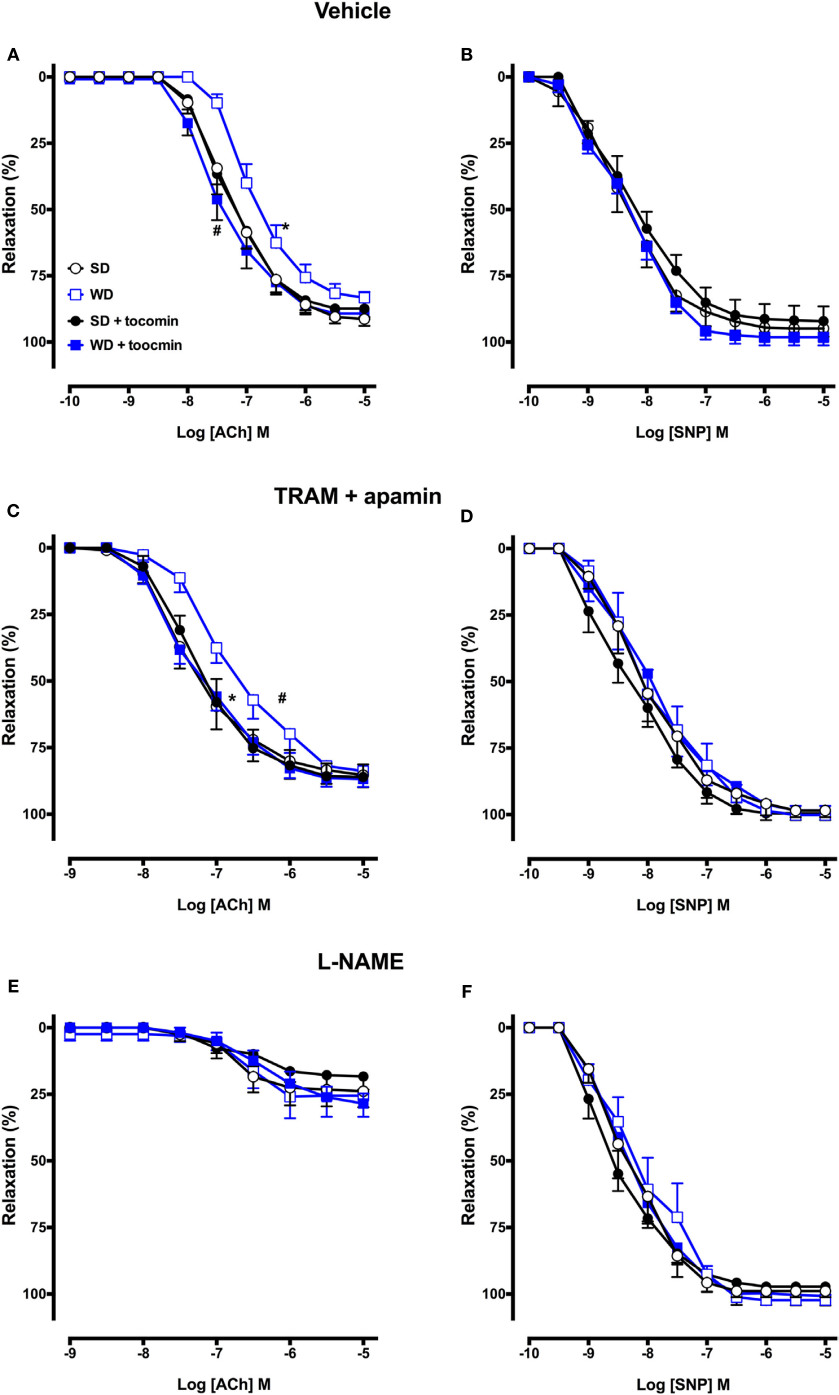
**Cumulative concentration–response curves to ACh and SNP in the absence or presence of tocomin in endothelium-intact aortae isolated from standard diet (SD), western diet (WD), and tocomin treated (SD + tocomin/WD + tocomin) rats in the presence of vehicle (A,B), TRAM + apamin (C,D), and l-NAME (E,F)**. *pEC_50_ significantly different to SD. ^#^pEC_50_ significantly different to WD *p* ≤ 0.05. *n* = 4–10. Two-way ANOVA Sidak’s post test. Results are shown as mean ± SEM. See Table 2 for values and statistical comparison.

The sensitivity (pEC_50_), but not the maximum response, to the endothelium-dependent dilator ACh was significantly reduced in the aortae from WD compared to SD rats (Table 2; Figure 3A). Responses to SNP were not affected by the WD (Figure 3B) indicating that the high-fat diet selectively impaired endothelium-dependent relaxation. The 4-week treatment of the WD rats with tocomin (40 mg/kg/day sc) significantly improved sensitivity to ACh in the aorta from WD-fed, but not SD-fed, rats. Tocomin treatment did not affect the endothelium-independent relaxation to SNP in any group.

**Table 2:** **The effect of 4-week tocomin (40 mg/kg/day sc) treatment on ACh-induced endothelium-dependent and SNP-induced endothelium-independent relaxation of aortae from rats fed a standard diet (SD) or high-fat western diet (WD)**.

		ACh			SNP	
		
	*n*	pEC_50_ (M)	*R*_max_ (%)	*n*	pEC_50_ (M)	*R*_max_ (%)
**Control**						
Standard diet	10	7.24 ± 0.10	91 ± 3	9	8.30 ± 0.17	95 ± 3
Western diet	8	6.85 ± 0.12^#^	83 ± 2	8	8.35 ± 0.10	98 ± 3
SD + tocomin	8	7.27 ± 0.11	89 ± 3	8	8.30 ± 0.13	92 ± 5
WD + tocomin	10	7.44 ± 0.12^$^	89 ± 2	8	8.35 ± 0.10	98 ± 3
**TRAM + apamin**						
Standard diet	10	7.24 ± 0.15	85 ± 5	9	7.99 ± 0.26	99 ± 2
Western diet	8	6.77 ± 0.16^#^	84 ± 2	6	7.62 ± 0.21	100 ± 3
SD + tocomin	8	7.19 ± 0.10	86 ± 5	8	7.95 ± 0.26	95 ± 4
WD + tocomin	9	7.27 ± 0.10^$^	87 ± 3	8	7.96 ± 0.24	98 ± 1
**l-NAME**						
Standard diet	7	6.47 ± 0.27	24 ± 5*	5	8.36 ± 0.21	99 ± 2
Western diet	7	6.70 ± 0.16	26 ± 8*	7	8.22 ± 0.24	102 ± 2
SD + tocomin	6	6.70 ± 0.22	11 ± 4*	8	8.40 ± 0.26	98 ± 3
WD + tocomin	9	6.16 ± 0.27	30 ± 4*	8	8.30 ± 0.13	101 ± 4
**l-NAME + ODQ**						
Standard diet	9	6.82 ± 0.23	8 ± 3*	5	5.27 ± 1.2*	27 ± 4*
Western diet	7	6.30 ± 0.11	17 ± 2*	4	6.37 ± 0.08*	70 ± 5*
SD + tocomin	9	6.97 ± 0.34	12 ± 6*	4	6.19 ± 0.64*	35 ± 14*
WD + tocomin	9	6.33 ± 0.23	18 ± 2*	6	6.54 ± 0.29*	63 ± 10*
**l-NAME + ODQ + TRAM + apamin**						
Standard diet	4	6.23 ± 0.58	8 ± 4*	4	5.59 ± 0.16*	29 ± 6*
Western diet	8	6.12 ± 0.11	13 ± 1*	6	6.00 ± 0.24*	64 ± 6*
SD + tocomin	8	6.41 ± 0.27	8 ± 3*	4	4.35 ± 1.1*	47 ± 8*
WD + tocomin	9	6.50 ± 0.22	7 ± 2*	8	6.21 ± 0.37*	63 ± 9*

*Significantly different to control (SD), *p* < 0.05.

*^#^Significantly different to SD of same treatment group, p < 0.05*.

*^$^Significantly different to WD of same treatment group, p < 0.05*.

*Sidaks multiple comparisons test*.

*Table illustrates the pEC_50_ and *R*_max_ values for ACh in aortic rings from standard diet (SD), western diet (WD), and tocomin (40 mg/kg/day sc) treated (SD + tocomin/WD + tocomin) groups. The effect of various inhibitors is shown. The data are expressed as mean ± SEM. *n* = no. of experiments*.

### Effect of Western Diet and Tocomin on NO-Mediated Relaxation

In the presence of inhibitors of SK_Ca_ (apamin) and IK_Ca_ (TRAM-34), ACh-induced relaxation is mediated by NO. The sensitivity to ACh was significantly decreased in the WD rat aorta compared to the SD aorta (Table 2; Figure 3C). Following 4-week treatment with tocomin, there was no change in ACh-induced relaxation in aortae from rats fed the SD, but the sensitivity to ACh was significantly increased in the WD rat aorta by the tocomin treatment. Tocomin treatment had no effect on the endothelium-independent relaxant responses to SNP in any group (Figure 3D). This suggests that tocomin treatment improves endothelial release of NO from the WD rat aorta rather than influencing sensitivity to either endogenous or exogenous NO.

### Effect of Western Diet and Tocomin on EDH-Type Relaxation

**Figure 4:**
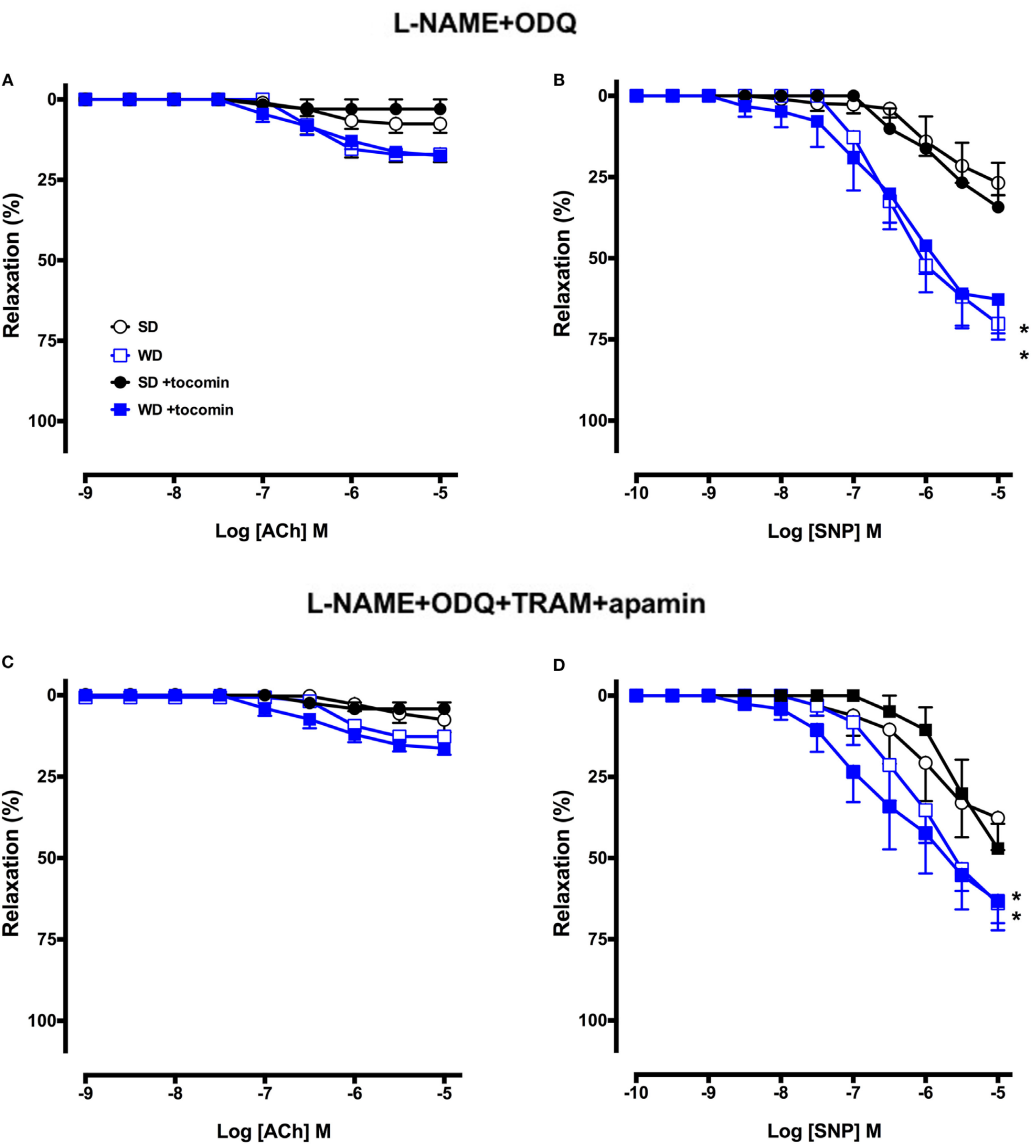
**Cumulative concentration–response curves to ACh and SNP in the absence or presence of tocomin in endothelium-intact aortae isolated from standard diet (SD), western diet (WD), and tocomin-treated (SD + tocomin/WD + tocomin) rats in the presence of l-NAME + ODQ (A,B) and l-NAME + ODQ + TRAM + apamin (C,D)**. *R_max_ significantly different to SD *n* = 4–8. Two-way ANOVA Sidak’s post test. Results are shown as mean ± SEM. See Table 2 for values and statistical comparison.

In the presence of the eNOS inhibitor l-NAME alone and in combination with the sGC inhibitor ODQ, the maximum relaxation to ACh was significantly decreased in SD rat aortae (Figure 4; Table 2), indicating the predominant contribution of NO to endothelium-dependent relaxation in this large artery. ACh-induced relaxation in the presence of l-NAME, with or without ODQ, was not different in the aortae from WD rats compared to SD rats (Table 2; Figures 3E and 4A), and tocomin did not affect responses in either groups. These results suggest that in both the SD and WD rat aortae eNOS derived NO is the main cause of endothelium-dependent relaxation and 4-week treatment of WD rats with tocomin does not improve ACh sensitivity through an eNOS/sGC-independent mechanism.

The presence of TRAM-34 and apamin, in addition to l-NAME and ODQ, did not further attenuate responses to ACh in any group (Figure 4C) indicating an absence of contribution of calcium-activated potassium (K_Ca_) channels to endothelium-dependent relaxation in this large artery.

### Effect of Western Diet and Tocomin on SNP-Induced Relaxation

l-NAME did not significantly affect responses to SNP, but addition of ODQ did significantly reduce the sensitivity and maximum response to SNP in aortae from both SD and WD rats (Table 2; Figures 3 and 4). Interestingly, in the presence of l-NAME and ODQ, SNP-induced relaxation was significantly greater in aortae from WD compared to SD rats. Tocomin did not affect responses to SNP under any of the tested conditions.

### The Effect of a Western Diet on Nox2, eNOS, and Modulatory Proteins

**Figure 5:**
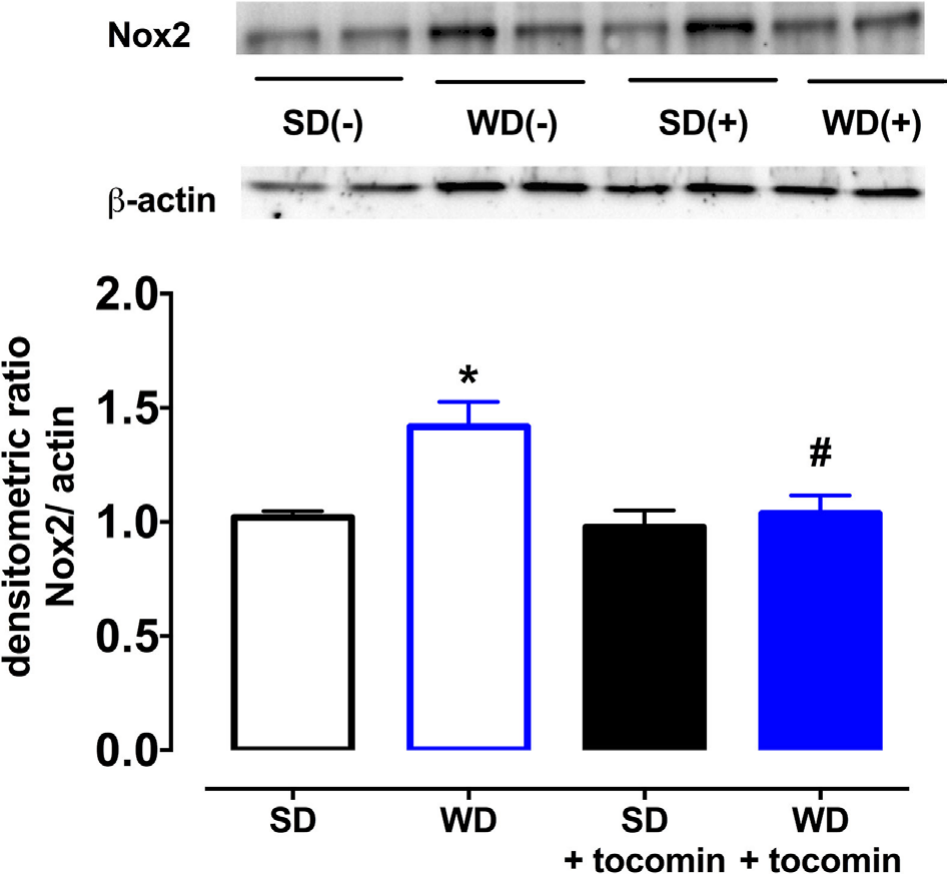
**Protein expression of NADPH oxidase (Nox-2) from isolated aortae from standard diet (SD), western diet (WD), and tocomin treated (SD + tocomin/WD + tocomin) rats**. Representative blots are shown for each corresponding graph. *Significantly different to SD. ^#^Significantly different to WD. Results are shown as means ± SEM; *n* = 6 experiments. *p* ≤ 0.05. Two-way ANOVA Dunnett’s post test.

The expression of the superoxide producing enzyme Nox2 was significantly increased in aortae from WD in comparison to the SD rats (Figure 5). Tocomin treatment did not affect Nox2 in aortae from SD rats but significantly reduced its expression in aortae from WD rats.

**Figure 6:**
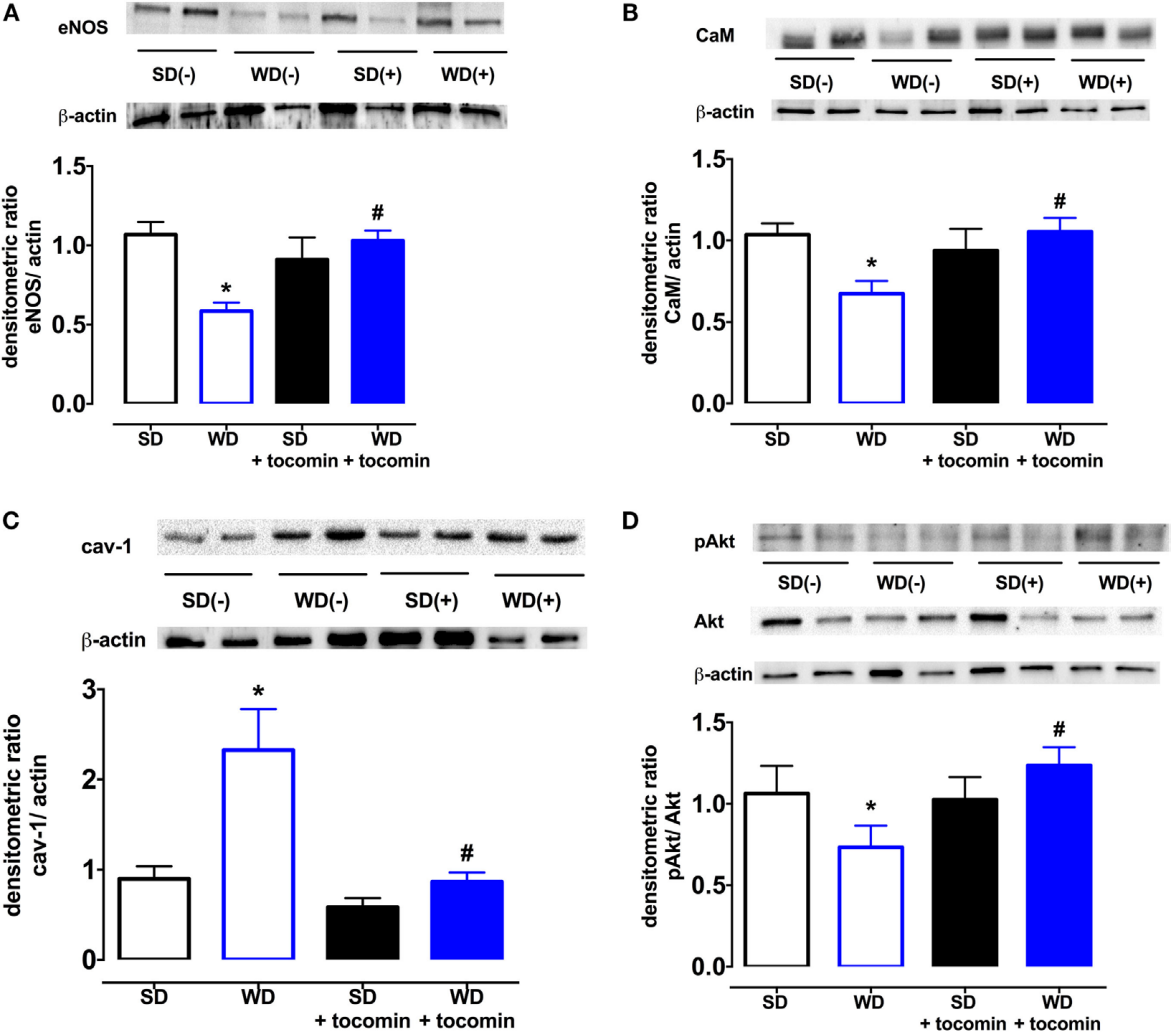
**Protein expression of total eNOS (A), calmodulin-1 (B), caveolin-1 (C), and pAkt/Akt (D) from isolated aortae from standard diet (SD), western diet (WD), and tocomin treated (SD + tocomin/WD + tocomin) rats**. Representative blots are shown for each corresponding graph. *Significantly different to SD. ^#^Significantly different to WD. Results are shown as means ± SEM; *n* = 6 experiments. *p* ≤ 0.05. Two-way ANOVA Dunnett’s post test.

The total expression of the NO producing enzyme eNOS was significantly lower in aortae from WD in comparison to SD rats. In addition, in WD rat, aortae expression of calmodulin (CaM) was decreased, and caveolin-1 was increased in comparison to SD rats. The WD also decreased the proportion of Akt that was phosphorylated (Figure 6). Treatment with tocomin reversed the diet-induced changes in eNOS, caveolin-1, CaM, and the phosphorylation of Akt (Figures 5 and 6).

## Discussion

The results of this study demonstrate that rats fed a high-fat, “western” diet exhibit an impairment of endothelium-dependent relaxation that is associated with an increased expression of the NADPH oxidase Nox2 subunit in the aorta and an increase in the vascular generation of superoxide. The increase in vascular oxidative stress was accompanied by a decrease in basal NO release and impairment of the contribution of NO to ACh-induced relaxation. A decreased expression of eNOS, calmodulin, and phosphorylated Akt and an increase in caveolin-1 are likely to contribute to the impaired NO-mediated relaxation. Tocotrienol-rich tocomin was able to prevent the diet-induced changes in vascular function, apparently due to an antioxidant action, a property of tocomin that we have previously demonstrated in isolated vascular tissue (11).

### Effect of Western Diet on Oxidative Stress and Endothelium-Dependent Relaxation

The high-fat WD did not cause any increase in blood glucose levels or HbA1c at the end of the study, but the WD group had a significantly higher body weight and epididymal fat mass at the end of the feeding period (Table 1) similar to observations previously made when using the same diet (16). Epididymal fat pad mass provides an established indication of obesity (17).

The WD caused a significant increase in the superoxide generated by the aortae and this correlated with an increase in the expression of the NADPH oxidase subunit Nox2. We and others have reported that diets high in fats and sugars cause oxidative stress in the cardiovascular system (3–5, 18, 19). Our observation that there was an increased expression of Nox2 that might contribute to the generation of vascular oxidative stress is similar to a previous report that a high-fat, high-sucrose diet increased vascular Nox2, but not Nox4, expression in mice (20). The WD-induced increase in oxidative stress was accompanied by a decreased contractile response to the NOS inhibitor l-NAME and an impaired response to the endothelium-dependent relaxant ACh without any effect on the NO donor SNP. These observations are indicative of a selective impairment of endothelial function in the rat aortae as has been previously reported (3, 4), when there is an increase in oxidative stress including in response to consumption of a high-fat diet (5, 21).

### Effect of Tocomin on Endothelial Function

Treating WD rats with tocomin did not affect the blood glucose or HbA1c levels nor did it decrease bodyweight or epididymal fat mass. However, there was a significant decrease of superoxide generation in arteries from WD, but not SD, fed rats and a decrease in the Nox2 expression, once again only in the WD fed rat aortae. Tocomin treatment did not affect responses to ACh or SNP in aortae from SD fed rats but did increase the sensitivity to ACh in the WD fed rat aortae. Thus tocomin, which we have previously reported to acutely enhance endothelium-dependent relaxation in isolated vascular tissue exposed to oxidative stress (11), is also able to improve endothelial function when administered *in vivo*.

### Effect of Tocomin on NO-Mediated Relaxation

We next investigated whether the NO-mediated component of the endothelium-dependent relaxation was influenced by the WD and/or by tocomin treatment by examining responses to ACh in the presence of the K_Ca_ channel blockers TRAM-34 plus apamin to eliminate the EDH-type component of relaxation. In the presence of TRAM-34 and apamin, the ACh-induced relaxation was significantly impaired in aortae from WD rats compared to SD rats indicating that the high-fat diet impaired NO-mediated relaxation. This impairment of responses to stimulated release of NO was consistent with the observation that the basal release of NO was also impaired in WD vessels, demonstrated by a decreased contractile response to NOS inhibition. A number of factors may have contributed to the WD-induced impairment of NO release. The expression of eNOS was significantly reduced by consumption of the high-fat diet. Further, the expression of caveolin-1, which inactivates eNOS, was increased, whereas calmodulin and pAkt expression was decreased. Calmodulin and pAkt both act to promote eNOS activity (22, 23). eNOS activity may also be influenced by the phosphorylation of Ser1177 and Thr495, among other key sites (24), but eNOS phosphorylation was not investigated in this study. The changes in modulatory protein expression are similar to changes observed under other circumstances of increased oxidative stress, such as diabetes (15, 25). It is also well established that oxidative stress may also cause uncoupling of eNOS to promote synthesis of superoxide rather than NO (24). We have previously reported that diabetes causes eNOS uncoupling (25) and, while we did not explore whether a high-fat diet has a similar effect in this study, obese Zucker rats are reported to have an elevated level of uncoupled eNOS (26). Treatment with tocomin significantly increased NO-mediated relaxation in WD aortae but had no effect on relaxation of SD vessels. Tocomin treatment caused several effects that might have contributed to the increased NO activity in the WD aortae. First, the decrease in Nox2 would contribute to the decrease in vascular superoxide generation reducing the inactivation of NO and formation of ONOO^−^. Furthermore, tocomin treatment increased expression of eNOS and calmodulin and increased phosphorylation of Akt to return levels in WD rats to be similar to those seen in SD rats. There was also a decrease in caveolin-1. Together, these actions are consistent with an increased capacity to synthesize NO reflected as both an improved basal release of NO, demonstrated by the contractile response to a NOS inhibitor, and enhanced ACh-induced relaxation reflecting stimulated NO release.

An interesting observation was that SNP, an endothelium-independent NO donor, caused a significantly greater relaxation in WD versus SD aortae when NOS and sGC were inhibited. This is a surprising observation and there appear to be no previous reports of studies where responses to a NO donor were examined under similar conditions. The data suggest that WD may increase the activity of sGC or impair the inhibitory capacity of ODQ. Further investigation would be required to investigate these possibilities. Tocomin treatment did not affect the response to SNP under any conditions. The ability of tocomin to enhance responses only to endogenous NO supports the importance of the treatment on improvement of the endogenous generation of NO through the increased expression of eNOS, calmodulin, and pAkt and the decreased expression of caveolin-1.

### Effect of Tocomin on EDH-Type Relaxation

To investigate whether the non-NO, EDH-type component of the endothelium-dependent relaxation was influenced by the WD and/or by tocomin treatment, we examined responses to ACh in the presence of the NOS inhibitor l-NAME and the sGC inhibitor ODQ. In this large artery from SD-fed rats, only a small response to ACh remained in the presence of l-NAME, and the addition of ODQ virtually abolished the remnant relaxation indicating that normally there was little if any role for EDH-type relaxation. Responses in aortae from WD rats were the same as in SD rats, and tocomin treatment did not change responses in any group indicating that there was no influence of the treatment on EDH-type relaxation. We have previously found that an antioxidant can improve NO but not EDH-type relaxation even in small arteries where non-NO mediators make a more marked contribution to relaxation than in the large artery used in this study (25). The small relaxation remaining in the presence of l-NAME, ODQ, TRAM, and apamin is most likely mediated by prostacyclin.

## Conclusion

There is a significant body of evidence gathered from both animal and human studies that ROS play a fundamental role in cardiovascular disease (27), including in disease associated with obesity (2). It is logical then that there has been intensive investigation of antioxidants in the prevention and treatment of cardiovascular disease, but despite many positive results in animal studies, the outcomes of large clinical trials have been predominantly disappointing (7). Studies examining the potential benefits of vitamin E have predominantly focused on α-tocopherol, one of eight naturally occurring isoforms of that vitamin, while there has been relatively little investigation of the potential capacity of tocotrienols as therapeutic agents (10). There is growing evidence that tocotrienols do not simply mimic the biological actions of tocopherols. For example, tocotrienols are reported to more effectively accumulate in cells compared to tocopherols, and this has been suggested to promote their efficacy as antioxidants (28). Further, we have previously reported that the combination of tocotrienols with α-tocopherol is better able to preserve endothelial function in the presence of oxidative stress than either tocopherol or tocotrienols alone (11). The outcome of the present study, where a tocotrienol-rich extract that includes some tocopherol preserves endothelial function in the presence of obesity-induced oxidative stress, suggests further investigation of tocomin as a potential therapeutic agent is warranted.

We have demonstrated that the tocotrienol-rich extract of palm oil, tocomin, increases NO activity to improve endothelium-dependent relaxation in aortae from rats fed a high-fat “western” diet. Tocomin did not affect the diet-induced weight gain or increase in epididymal fat but did attenuate the vascular oxidative stress. Our previous study demonstrated that tocomin, which contains a high proportion of tocotrienols (48%) with some α-tocopherol (11%), is able to acutely reduce oxidative stress to improve endothelium-dependent relaxation *in vitro* (11). An additional beneficial action in this study to reduce vascular oxidative stress was a decreased expression of the vascular NADPH oxidase subunit Nox2. A further positive outcome of tocomin treatment in the obese rats was an increased expression of eNOS and the eNOS activity promoting proteins calmodulin and pAkt. Further, there was also a decreased expression of the inhibitory protein caveolin-1. The beneficial actions of tocomin in this diet-induced model of obesity suggest that it may have potential to be used as a therapeutic agent to prevent vascular disease in obesity.

## Author Contributions

SA and JN conducted the experiments. SA, JN, TJ, and OW developed the experimental plan and contributed to the writing of the manuscript. SA and OW undertook data analysis.

## Conflict of Interest Statement

The authors declare that the research was conducted in the absence of any commercial or financial relationships that could be construed as a potential conflict of interest.
